# Effectiveness of Aqueous Chlorine Dioxide in Minimizing Food Safety Risk Associated with *Salmonella*, *E. coli* O157:H7, and *Listeria monocytogenes* on Sweet Potatoes

**DOI:** 10.3390/foods9091259

**Published:** 2020-09-08

**Authors:** Phillip Luu, Vijay Singh Chhetri, Marlene E. Janes, Joan M. King, Achyut Adhikari

**Affiliations:** School of Nutrition and Food Sciences, Louisiana State University Agricultural Center, Baton Rouge, LA 70803, USA; pluu2@lsu.edu (P.L.); vijayschhetri@gmail.com (V.S.C.); MJanes@agcenter.lsu.edu (M.E.J.); jking@agcenter.lsu.edu (J.M.K.)

**Keywords:** chlorine dioxide, sweet potatoes, *Salmonella*, *Escherichia coli* O157:H7, *Listeria monocytogenes*

## Abstract

Sodium hypochlorite (NaOCl) is a commonly used sanitizer in the produce industry despite its limited effectiveness against contaminated human pathogens in fresh produce. Aqueous chlorine dioxide (ClO_2_) is an alternative sanitizer offering a greater oxidizing potency with greater efficacy in reducing a large number of microorganisms. We investigated the effect of aqueous chlorine dioxide treatment against human pathogens, *Salmonella*, *Escherichia coli* O157:H7, and *Listeria monocytogenes* seeded on sweet potatoes. Sweet potatoes were spot inoculated (4.2 to 5.7 log CFU/cm^2^) with multi-strain cocktails of *Salmonella* spp., *E. coli* O157:H7, and *L. monocytogenes* and treated for 10–30 min with 5 ppm aqueous ClO_2_ or water. Aqueous ClO_2_ treatment was significantly (*p* < 0.05) effective in reducing *Salmonella* with a reduction of 2.14 log CFU/cm^2^ within 20 min compared to 1.44 log CFU/cm^2^ for water treatment. Similar results were observed for *L. monocytogenes* with a 1.98 log CFU/cm^2^ reduction compared to 0.49 log CFU/cm^2^ reduction observed after 30 min treatment with aqueous ClO_2_ the water respectively. The maximum reduction in *E. coli* O157: H7 reached 2.1 Log CFU/cm^2^ after 20 min of treatment with aqueous ClO_2_. The level of the pathogens in ClO_2_ wash solutions, after the treatment, was below the detectable limit. While in the water wash solutions, the pathogens’ populations ranged from 3.47 to 4.63 log CFU/mL. Our study indicates that aqueous ClO_2_ is highly effective in controlling cross-contamination during postharvest washing of sweet potatoes.

## 1. Introduction

Growing global populations have greatly increased the demand of wholesome fresh produce including sweet potatoes. The global sweet potato market has been on the rise since 2012, producing 105.2 million tons of the crop and yielding 1.4 million tons in 2016 in the United States alone [1]. Sweet potatoes are recognized as nutritiously potent crops consumed and researched globally [2,3]. The sweet potato is potentially an ideal and efficient crop for providing sustenance for much of the world as well as being a viable crop for sustainable agriculture [2,4]. The majority of sweet potatoes are produced by developing countries where the usage of raw manure as fertilizer is still common practice. In addition to pathogens found in raw manure, contaminated irrigation water and wildlife excrements pose potential threats to soil contamination.

Fresh produce commodities have been subject to numerous foodborne outbreaks in both the domestic and international markets [5]. With cross-contamination vectors including soil and irrigation water contamination as well as produce handling by field workers, postharvest sanitation is often the most vital step in reducing microbial contamination [6,7,8]. Fresh-cut produce sanitation has been well-researched. Few studies have highlighted the importance of postharvest sanitation of root crops. Root crops tend to be overlooked in favor of minimizing cross-contamination in fresh produce that are consumed raw. However, crops grown under the soil are vulnerable to harboring pathogens as a result of poor compost practices and the usage of contaminated irrigation water [9]. Despite cooking being an acceptable kill step in eliminating most pathogens, root crops such as sweet potatoes and potatoes that are typically cooked in sealed aluminum foil, an anaerobic environment, are susceptible to botulism as a result of *C. botulinum* spores germinating during the baking process [10,11]. Additionally, root vegetables such as carrots, beets, radishes, and sweet potatoes may also be eaten raw, which may pose a food safety risk.

Produce are washed to remove excess dirt and debris from surfaces. However, without a sanitizing agent in the wash solution, washing will have minimal impact on reducing bacterial populations [12,13]. Sweet potatoes are sanitized in dump tanks using 100–150 ppm of sodium hypochlorite (NaOCl) [14]. However, the efficacy of NaOCl diminishes as greater amounts of organic materials build up in the sanitizing solution [15]. Pathogen control is also reliant on maintaining a narrow pH range [16]. In addition, the chlorine treatment can potentially produce carcinogenic compounds, such as trichloramines, as byproducts of the treatment posing public health risks [17,18]. Chlorine dioxide (ClO_2_) has gained popularity within the last couple of decades to disinfect drinking water and wastewater, and to sanitize fresh produce and poultry [19]. ClO_2_ has 2.5 times the oxidizing capability as Cl_2_ and may be an effective alternative produce sanitizer [20]. ClO_2_ offers similar sanitation efficacy as chlorine-based sanitizers while requiring far less concentration in a wide pH range (3–8) [21]. Additionally, ClO_2_ does not have issues related to the production of carcinogenic byproducts [22].

Previous studies have demonstrated the efficacy of ClO_2_ in reducing human pathogens including Shiga-toxin-producing *Escherichia coli*, *Salmonella enterica*, *Listeria monocytogenes*, *Pseudomonas aeruginosa*, *Staphylococcus aureus*, and *Yersinia enterocolitica* on a variety of produce [19]. The microbial death is believed to be due to oxidation of sulfhydryl groups on cell-surface proteins and increased permeability of the outer membrane [23]. Another mechanism is its influence on internal components of the cells such as proteins and nucleic acids as well as interfering protein synthesis [19,24]. At 5 ppm, aqueous ClO_2_ has demonstrated cross-contamination control of *Salmonella*, *Escherichia coli* O157:H7, and *Erwinia carotovora* in washing produce [16,25]. Blueberries sanitized with 5 ppm aqueous ClO_2_ were found to have 2.24 log CFU/g reduction in *L. monocytogenes* after 30 min of treatment with further treatment time resulting in similar levels of reduction [26]. Green peppers exhibited a 6.45 log CFU/g reduction in *E. coli* O157:H7 after 30 min of 1.24 mg/L aqueous ClO_2_ treatment [13]. The objective of this study was to evaluate the efficacy of aqueous chlorine dioxide on reducing bacterial pathogens (*E. coli* O157:H7, *Salmonella* spp., and *L. monocytogenes*) from sweet potatoes and its role in minimizing cross-contamination during washing.

## 2. Materials and Methods

### 2.1. Produce Material

Fresh Beauregard sweet potatoes (*Ipomoea batatas*) sourced from Black Gold farms in Delhi, Louisiana were held at 4 °C for no longer than 12 weeks. Freshly harvested sweet potatoes collected before the curing step, without removing the surface soil were also used to examine the difference between the cured and uncured samples. Sweet potatoes with average surface areas of 274–293 cm^2^ were selected for the experiment.

### 2.2. Bacterial Strains

Several strains of *S. enterica*, *E. coli* O157:H7, *L. monocytogenes* derived from outbreaks as well as a nonpathogenic strain of *Enterococcus* spp., were used in this study. These pathogenic strains were generously supplied by Dr. Michelle D. Danyluk at the University of Florida. A cocktail of *Salmonella enterica* (Anatum strain 1715a, Enteritidis PT 30, and Enteritidis PT 9c Strain RM4635), *E. coli* O157:H7 (Odwalla strain 223, CDC 658, and H1730), and *Listeria monocytogenes* (101M serotype 4b, Scott A serotype 4b, and V7 serotype 1/2a) were used in this study. All serotypes were routinely grown in tryptic soy broth (TSB) (Hardy Diagnostics, Santa Maria, CA) at 37 °C for 24 h. The bacterial cultures were stored in glycerol (70:30, vol/vol, culture: glycerol) at −80 °C prior to usage.

### 2.3. Preparation of Inoculum

Each frozen culture was activated by three consecutive culture transfers (24 h each) to respective broth maintaining 37 °C [27]. The TSB (BD Difco, Sparks, MD, USA) was used for the activation of *S. enterica* and *E. coli* O157:H7 and TSB with 0.6% yeast extract (BD Difco, Sparks, MD, USA) for *L. monocytogenes*. The cocktail of pathogens was prepared by mixing 10 mL of each serotype broth into 50 mL centrifuge tubes and vortexed for 2 s. Cells were harvested via centrifugation (Allegra X-15R, Beckman Coulter, Indianapolis, IN, USA) at 6500 rpm for 5 min and the supernatant was decanted. Cell pellets were washed in 10 mL of sterile 1× phosphate buffer saline (PBS) (Hardy Diagnostics, Santa Maria, CA, USA), centrifuged at 6500 rpm for 5 min, and resuspended in 5 mL of sterile 1× PBS. To prepare the inocula, the cell suspension cocktails were diluted and adjusted to approximately 10^8^–10^9^ CFU/mL.

### 2.4. Inoculation of Sweet Potatoes

Sweet potatoes were spot inoculated with 500 µL of the inoculum. The inoculum was gradually introduced at a rate of 100 µL aliquots on the upper surface of the sweet potatoes to minimize inoculum loss from runoff. Sweet potatoes were then air-dried for 1.5 h inside a biosafety cabinet to allow for bacterial attachment. During the study, the initial pathogen level on inoculated sweet potatoes was 4.2 to 5.7 log CFU/cm^2^.

### 2.5. Preparation of Aqueous ClO_2_

Sodium chlorite solution (450 mL of 10 μg/μL) was mixed with 1N hydrochloric acid (21 mL) in a 1000 mL PYREX^®^ storage bottle and stored for 1 h at room temperature with continuous stirring [28,29]. The final working concentration of 5 ppm was prepared by diluting the stock solution with distilled water. The concentration was confirmed by direct-reading method (HACH DR 900, Loveland, CO, USA). The pH value of the ClO_2_ solutions averaged at 8.67.

### 2.6. Aqueous ClO_2_ Treatment

Sweet potatoes inoculated with 4.2 to 5.7 log CFU/cm^2^ of *S. enterica*, *E. coli* O157:H7, or *L. monocytogenes* and noninoculated sweet potatoes were treated with aqueous ClO_2_ (5 ppm) or distilled water (control) for 10, 20, and 30 min at 22 ± 1 °C. Briefly, sweet potatoes were placed in sterile polypropylene bags (VWR^®^, Radnor, PA, USA) containing the treatment solutions. The bags were then sealed to minimize ClO_2_ concentration loss and were placed in buckets. Each sample was agitated for 30 s to ensure treatment contact on sweet potato surfaces.

### 2.7. Recovery of Pathogens and Microbiological Analyses

Three additional controls that received no treatment were used in this study. The first control was conducted with a duplicate of noninoculated, untreated sweet potatoes used to detect background microbes and potential pathogens. The second control was conducted with two duplicates of inoculated, untreated sweet potatoes used to determine the initial pathogen levels of treated samples. The third control was conducted with a duplicate of inoculated, untreated sweet potatoes used to determine the surviving pathogen levels following the end of the treatments. Treatment solutions from aqueous ClO_2_ and water treatments were sampled for microbiological analysis. After treatment, inoculated and noninoculated sweet potato samples were aseptically transferred into a stomacher bag (Nasco Whirl-pak, Fort Atkinson, WI, USA) with 100 mL sterile 0.1% peptone water (Hardy Diagnostics, Santa Maria, CA, USA). Samples were gently massaged for 2 min to dislodge attached pathogens. Serial 10-fold dilutions were prepared in 0.1% peptone water. Dilutions were spread plated in duplicates on selective media with Xylose Lysine Deoxycholate agar (XLD) (BD Difco, Sparks, MD, USA) for *S. enterica*, Sorbitol MacConkey Agar (SMAC) (BD Difco, Sparks, MD, USA) supplemented with Cefixime-Tellurite Supplement (CT) (HiMedia Laboratories, Mumbai, India) for *E. coli* O157:H7, and Oxford Agar base (BD Difco, Sparks, MD, USA) for *L. monocytogenes*. Plates were incubated at 37 °C for 24 h. Enumerated colony results were expressed as log CFU/cm^2^.

### 2.8. Statistical Analysis

The pathogen populations (CFU) recovered from sweet potatoes and treatment solutions were converted to log CFU/cm^2^ or ml. Each experiment was performed three times independently. The data was analyzed by ANOVA test using Statistical Analysis Software (SAS) with Tukey’s post hoc test to determine mean differences (*p* < 0.05) (SAS Institute, Cary, NC, USA).

## 3. Results

### 3.1. Effects of Aqueous ClO_2_ Treatment on Salmonella Enterica

**Figure 1:**
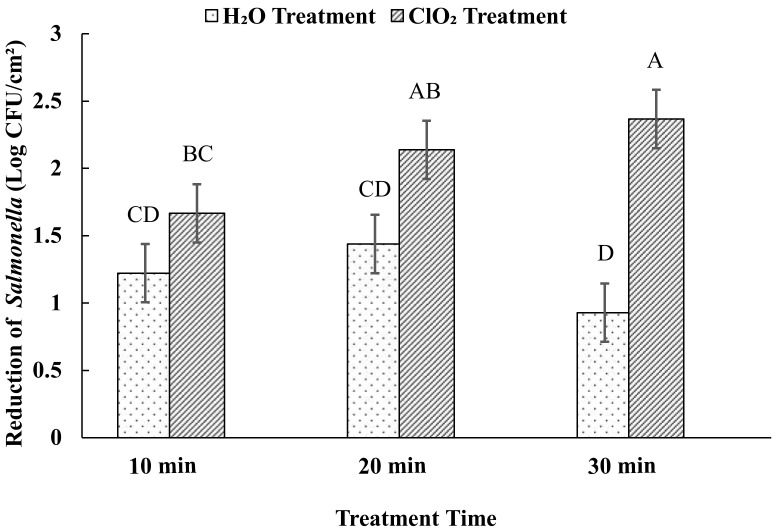
Effects of aqueous ClO_2_ and water treatment on the reduction of *Salmonella* spp. Reduction of *Salmonella* spp. seeded on sweet potato surfaces was evaluated after 10, 20, and 30 min of aqueous ClO_2_ (5 ppm) and water treatments at 22 ± 1 °C. Each bar diagram represents average counts ± standard error. Different uppercase letters on the top of the diagrams means that the reductions between the treatments and times were significantly different (*p* < 0.05).

The effect of 5 ppm aqueous ClO_2_ treatment on reducing *Salmonella* populations inoculated on sweet potato surfaces was investigated with treatment times ranging from 10 to 30 min (Figure 1). The level of reduction in *S. enterica* population increased with an increase in exposure time. The level of reduction was 1.67, 2.14, and 2.37 log CFU/cm^2^ at 10, 20, and 30 min of exposure, respectively. Water resulted in the reduction of the population by 1.22 log CFU/cm^2^ at 10 min. However, no significant reduction was observed with an increase in exposure time with water, up to 30 min.

The disinfectant efficacy of aqueous ClO_2_ could be higher in water. The treatment with 5 ppm ClO_2_ resulted in the reduction of *S. enterica* level by ≥5-log within 6 s [30]. However, the efficacy of the sanitizer is drastically reduced on produce surfaces. Although complete elimination of pathogens from fresh produce is not possible using this sanitizer at acceptable concentration, its use in produce industry may help minimize produce safety risks by reducing the pathogens level and controlling cross-contamination between the products and the batches [22,30,31]. To our knowledge, there are no published findings on the sanitizing efficacy of aqueous ClO_2_ specifically on sweet potatoes. However, some studies have been published on other fruits and leafy vegetables. In a study with iceberg lettuce inoculated with *S. typhimurium*, similar results were observed after 10 min treatment with aqueous ClO_2_ [31]. Pathogen levels were reduced by 1.64 log CFU/g after the treatment with continuous mild agitation. Likewise, blueberries inoculated with *S. typhimurium* exhibited similar levels of reduction (1.93 log CFU/g) after 20 min of 5 ppm aqueous ClO_2_ treatment [26]. On apples, a total of up to 2 log reduction in *Salmonella* population was observed after 10 min of treatment with a similar concentration of the sanitizer [32]. Cherry tomatoes inoculated with *S. typhimurium* exhibited a 2.53 log CFU/g reduction after a 5 min treatment using 10 ppm aqueous ClO_2_ [33]. The treatment with 15 ppm of ClO_2_ resulted in the reduction of *S. typhimurium* by 3.32 log in 20 min on blueberry samples [26]. The treatment was more efficient on tomatoes samples. ClO_2_ of 20 ppm reduced *S. enterica* population by 5 log in 1 min [30]. While mungbean sprout required 100 ppm to eliminate the *S. typhimurium* by 3 log in 5 min [34]. The variations in the level of reductions between the studies can be attributed to differences in aqueous ClO_2_ concentrations and types of produce. The efficacy of the treatment increased with increase in the concentration of the aqueous ClO_2_ [31]. Our results indicated that, unlike water treatment, an increase in the ClO_2_ treatment time increased the level of reduction in *Salmonella* populations, with more than 2 log reduction after 20 min of exposure.

### 3.2. Effects of Aqueous ClO_2_ Treatment on E. coli O157:H7

**Figure 2:**
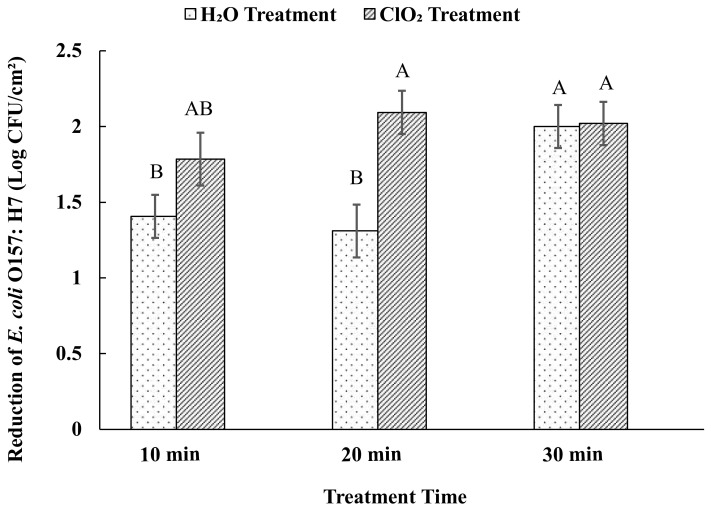
Effects of aqueous ClO_2_ and water treatment on the reduction of *E. coli* O157:H7. Reduction of *E. coli* O157:H7 on sweet potato surfaces after aqueous ClO_2_ treatment (5 ppm) for 10, 20, and 30 min and water treatment at 22 ± 1 °C. Each bar diagram represents average counts ± standard error. Different uppercase letters on the top of the diagrams means that the reductions between the treatments and times were significantly different (*p* < 0.05).

The effect of 5 ppm aqueous ClO_2_ on *E. coli* O157:H7 on sweet potato surfaces was investigated with treatment times ranging from 10 to 30 min (Figure 2). The ClO_2_ treatment for 10 min resulted in the reduction of the population by 1.78 log CFU/cm^2^. At 20 min of treatment, the reduction was by 2.0 log CFU/cm^2^, which was significantly higher than water (1.31 log CFU/cm^2^). However, after 30 min, water had a similar level of reduction as compared to ClO_2_.

Some studies showed similar levels of reduction in *E. coli* O157: H7 populations on produce surfaces due to ClO_2_ and water washing. It was reported that water washing for 30 min reduced *E. coli* O157:H7 levels by up to 2.4 log CFU on green peppers [13]. Iceberg lettuce leaves inoculated with *E. coli* O157:H7 resulted in 1.98 and 1.46 log CFU/g reduction after 10 min of 5 ppm aqueous ClO_2_ and distilled water treatment, respectively [31]. Another study observed the reduction of the population of this pathogen by 1.2 log after 5 min of treatment with ClO_2_ of 10 ppm [35]. Increase in the concentration of the sanitizer to 20 ppm and the time to 15 min resulted in the reduction by 1.7 log [36]. On apples, the reduction was around 1 log after the treatment with 5 ppm for 10 min [32]. However, the effect of treatments could be variable with the type of produce and their surface characteristics. Higher levels of reductions were observed on (~6 log CFU/g) apples, whole lettuce, strawberries, and cantaloupes by 5 ppm of aqueous ClO_2_ with exposure for 5 min [37]. The water washing resulted in the reduction of *E. coli* O157:H7 levels by up to 1.7 log CFU on injured surfaces and by up to 2.4 log CFU on uninjured green pepper surfaces [13]. The sweet potatoes used in this study were free of lesions, injuries, and scars. Further study using sweet potatoes with injured surfaces and with different surface characteristics could help to better understand the variability in the sanitizing efficacy of aqueous ClO_2_ specifically on sweet potatoes.

### 3.3. Effects of Aqueous ClO_2_ Treatment on Listeria Monocytogenes

**Figure 3:**
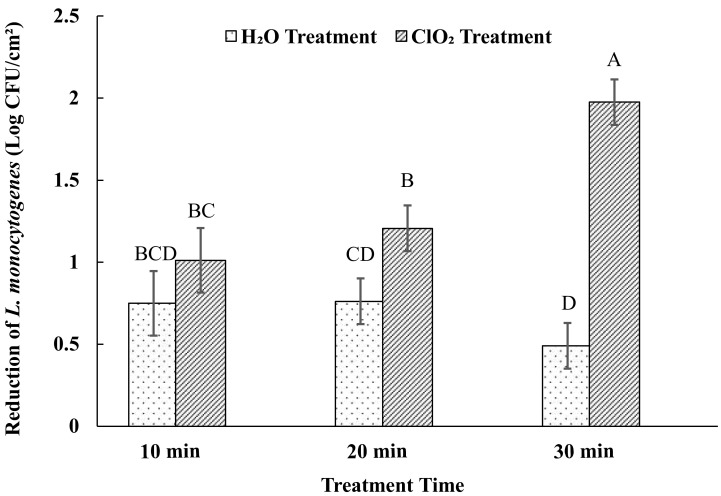
Effects of aqueous ClO_2_ and water treatment on the reduction of *L. monocytogenes.* Reduction of *L. monocytogenes* on sweet potato surfaces after 5 ppm aqueous ClO_2_ treatment for 10, 20, and 30 min and water treatment at 22 ± 1 °C. Each bar diagram represents average counts ± standard error. Different uppercase letters on the top of the diagrams means that the reductions between the treatments and times were significantly different (*p* < 0.05).

Reduction of *L. monocytogenes* on sweet potato surfaces increased with time, up to 30 min (Figure 3). Aqueous ClO_2_ treatment at 30 min had the greatest effect in reducing *L. monocytogenes* (1.98 log CFU/cm^2^). Although there was a higher reduction on ClO_2_ treated samples compared to the controls (water treated) at 10 min, a significantly higher reduction on those samples was observed only after 20 min. Similar to the effect on *Salmonella*, there was no significant difference between reductions due to water treatment among the three treatment times.

In a study with iceberg lettuce leaves contaminated with *L. monocytogenes*, reductions from approximately 7 log CFU/g to 5.36 log CFU/g were observed within 10 min of 5 ppm aqueous ClO_2_ treatment with light and continuous agitation [31]. This finding closer resembles the *L. monocytogenes* reduction achieved in our study after 20 min of treatment. As only 30 s of agitation was applied to treated samples at the start of the treatment, greater reduction may have been exhibited had continuous agitation been incorporated. Uninjured green peppers spot inoculated with *L. monocytogenes* observed 3.7 log reduction after 30 min of 3 ppm aqueous ClO_2_ treatment, far greater than our 30 min treatment result of 1.98 log CFU/cm^2^ [38]. In the same study, uninjured green pepper surfaces observed a 1.4 log reduction of *L. monocytogenes* populations after 30 min of water washing. However, our findings after 30 min of water washing (0.49 log CFU/cm^2^) showed more similarities to the 0.4 log reduction observed on injured green pepper surfaces [38]. The efficacy of the ClO_2_ treatment was different between the studies. The treatment of cabbage and lettuce with 5 ppm of ClO_2_ for 10 min resulted in the reduction of this pathogen by 0.8 log [39]. On blueberry samples, ClO_2_ of 15 ppm reduced the population by 4.88 log after 120 min of exposure [26]. While on mungbean sprouts, ClO_2_ of 100 ppm could reduce the population by 1.5 log after 5 min of treatment [34]. The differences in the efficacy between the studies may be attributed to the type of the produce used, the condition of the produce, bacterial strains and the study design. Some studies have already demonstrated that the type of pathogen or the strain could be one of the important factors influencing the efficacy of the sanitizers [22,30,31]. Surface properties of produce also has been found to have an influence on the efficacy of the sanitizers. Surface hydrophobicity was found to be an important factor in determining the inactivation of *E. coli* O157:H7, *S. typhimurium*, and *L. monocytogenes* by ClO_2_ gas [40].

### 3.4. Influences of Aqueous ClO_2_ on Cross Contamination of Noninoculated Sweet Potatoes

**Figure 4:**
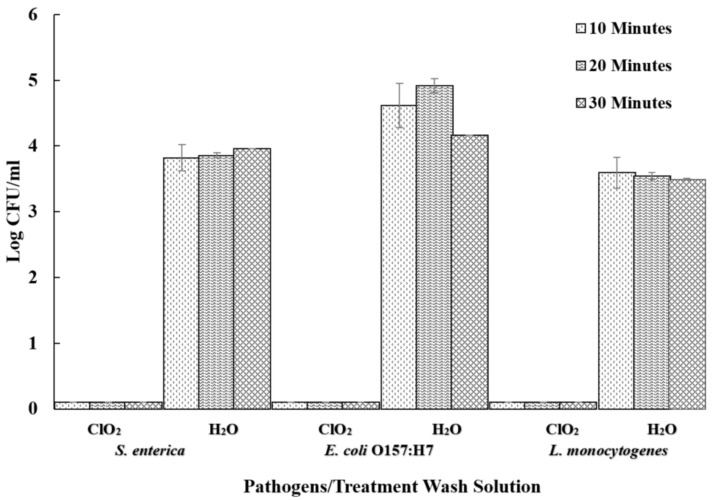
The recovery of pathogens from wash water. Pathogens recovered from wash solution following aqueous ClO_2_ treatment (5 ppm) for 10, 20, and 30 min and water treatment at 22 ± 1 °C.

The ClO_2_ was extremely effective in controlling bacterial levels from wash water (Figure 4). Pathogens from aqueous ClO_2_ treatment wash solutions were below the detectable limit of the test following 10, 20, and 30 min treatments. However, pathogens were detected up to 4.92 log CFU/mL from wash water without ClO_2_. Water treatment wash solutions recovered the greatest pathogen population from 4.16 to 4.92 log CFU/mL for *E. coli* O157:H7. Water treatment wash solutions from treating *Salmonella* and *L. monocytogenes* recovered 3.81 to 3.96 log CFU/mL and 3.49 to 3.59 log CFU/mL, respectively. These results suggest that using ClO_2_ during the washing of sweet potatoes can significantly reduce the risk of cross-contamination. In addition, ClO_2_ concentrations were below 3 ppm on sweet potatoes after 30 min of treatment, meeting the maximum residual surface concentration approved by the United States Food and Drug Administration (FDA) [41].

Use of ClO_2_ resulted in no recovery of the pathogens on noninoculated sweet potatoes when washed with inoculated samples (Table 1). Our results concurred with the findings reported by other studies. No recoverable *E. coli* O157:H7, *Salmonella enterica* and *S. typhimurium* was observed from noninoculated red chard baby leaves washed together with inoculated red chard baby leaves in 3 ppm aqueous ClO_2_ [16]. Similarly, 5 ppm aqueous ClO_2_ prevented the cross-contamination of tomatoes [25]. However, water wash contributed to the cross-contamination of noninoculated sweet potatoes by up to 3.40, 2.97 and 3.47 log CFU/cm^2^ of *Salmonella*, *E. coli* O157: H7 and *L. monocytogenes*, respectively (Table 1). In another study, wash water contaminated with 6.7 log CFU/mL of *Salmonella* spp. and 5.5 log CFU/mL of *Erwinia* spp., transferred 4.1 and 2.8 log CFU/cm^2^ of *Salmonella* spp. and *Erwinia* spp., respectively to tomatoes within 1 min of washing [25]. The ClO_2_ of 5 ppm was able to prevent the cross-contamination of tomatoes with *Salmonella* and *Erwinia* [30]. The results indicated that using ClO_2_ as low as 5 ppm could significantly reduce the risk of cross-contamination during postharvest washing.

### 3.5. Effects of Aqueous ClO_2_ on E. coli O157: H7 and Salmonella on Freshly Harvested Sweet Potatoes

**Figure 5:**
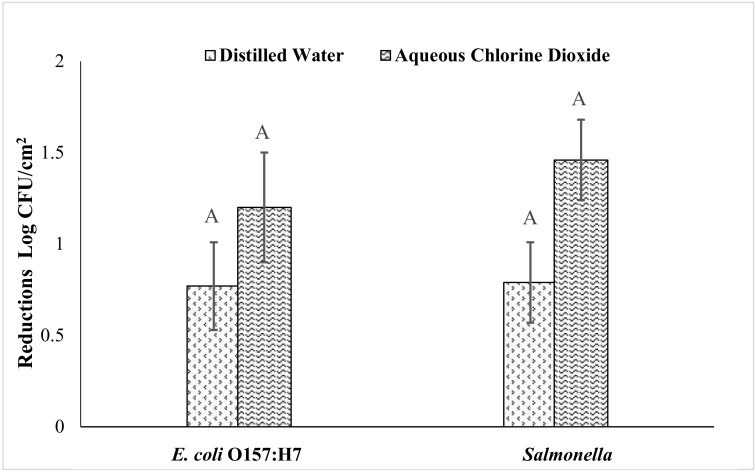
Effects of aqueous ClO_2_ and water treatment on the reduction of *E. coli* O157: H7 and *Salmonella* on freshly harvested sweet potatoes. Reduction of *E. coli* O157: H7 and *Salmonella* on freshly harvested sweet potatoes after aqueous ClO_2_ treatment (5 ppm) and water treatment for 10 min at 22 ± 1 °C. The data are presented as average counts ± standard error. Same uppercase letters on the top of the diagrams indicate no significant difference (*p* < 0.05) in between the treatments.

The effect of ClO_2_ and water treatment on *E. coli* O157: H7 and *Salmonella* inoculated on the freshly harvested local sweet potatoes is shown in Figure 5. As the sweet potatoes were collected before the curing step, the level of soil on them was higher than on the samples used in previous studies. The effect of the treatments on reducing the level of *Salmonella* and *E. coli* O157: H7 on these fruits was lower compared to on cured sweet potatoes. The aqueous ClO_2_ treatment for 10 min reduced the level of *Salmonella* and *E. coli* O157: H7 by 1.46 and 1.20 log CFU/cm^2^, respectively. The effect of water treatment was by 0.79 and 0.77 log CFU/cm^2^, respectively, which was not significantly different from the ClO_2_ treatment.

This study was aimed to consider freshly harvested sweet potatoes with higher levels of soil and bruises on the surfaces. Mechanical harvesting may result in bruises on the surfaces. As sweet potatoes we used in this study skipped the curing step, if there were bruises on these fruits, there was not enough time for the healing [42]. The injuries on produce surfaces may reduce the efficacy of the sanitizers [22]. Aqueous ClO_2_ (3 ppm) reduced the *L. monocytogenes* population by 3.7 log CFU when bell pepper surfaces were not injured and by 0.44 when injured [38]. Reduced efficacy of the treatments in our study may be due to a higher level of bruises and attached soiled particles which may have allowed bacterial cells to hide better in the surfaces limiting the access to the sanitizer solutions [43,44]. However, a further rigorous study is needed to better understand the influence of individual factors on the efficacy of the sanitizer.

The efficacy of the treatment has been increased by the combination of other strategies. The combination of aqueous ClO_2_ and fumaric acid increased the efficacy of the treatment against *E. coli* O157:H7, *S. typhimurium*, and *L. monocytogenes* inoculated on broccoli sprouts [45]. The combined treatments of ClO_2_ and ultrasound was found to have a role in maintaining postharvest storage quality of plum fruit [46]. Use of ultraviolet-C along with ClO_2_ was also effective in inhibiting microorganisms and maintaining shelf-life of fresh produce [47]. Another study evaluated the efficacy of ClO_2_ plus chitosan coating and found that this strategy could maintain the quality of fresh-cut bamboo shoot and extend their postharvest life [48]. These strategies had no significant effect on the sensory quality of the products. Furthermore, storage of mungbean sprouts using atmosphere packaging (MAP) followed by the treatment reduced the microbial population [34]. These results indicated that there is a room for the improvement with the treatment by combining other strategies even for sweet potatoes.

Overall, the application of chlorine dioxide at 5 ppm significantly reduced the pathogen’ levels during the postharvest washing of sweet potatoes. In addition, aqueous ClO_2_ can control cross-contamination in wash solutions, reducing the risk of cross-contamination of pathogens to uncontaminated sweet potatoes. The final concentration of ClO_2_ in sweet potatoes was below 3 ppm after 30 min of treatment, meeting the maximum residual surface concentration approved by the FDA [41]. Future research should incorporate longer and more vigorous agitation during treatment to better simulate conditions in processing facilities as well as sensory studies to evaluate whether aqueous chlorine dioxide at 5 ppm affects the desirable sensory qualities of sweet potatoes.

## Figures and Tables

**Table 1:** Cross-contamination of pathogens on noninoculated sweet potatoes washed together with inoculated sweet potatoes with or without aqueous chlorine dioxide.

Pathogen	Treatment Time	Recovery Average of Pathogens (log CFU/cm^2^)	
		Aqueous ClO_2_	Water Treatment
*Salmonella*	10	ND	3.40 ± 0.0
	20	ND	2.84 ± 0.2
	30	ND	2.84 ± 0.1
*E. coli* O157:H7	10	ND	2.97 ± 0.7
	20	ND	2.95 ± 0.5
	30	ND	2.38 ± 0.3
*L. monocytogenes*	10	ND	3.00 ± 0.6
	20	ND	3.18 ± 0.5
	30	ND	3.47 ± 1.1

ND = Not Detectable.
